# Novel Nano-Materials and Nano-Fabrication Techniques for Flexible Electronic Systems

**DOI:** 10.3390/mi9060263

**Published:** 2018-05-28

**Authors:** Kyowon Kang, Younguk Cho, Ki Jun Yu

**Affiliations:** School of Electrical Engineering, Yonsei University, Seoul 03722, Korea; kyowon.kang@yonsei.ac.kr (K.K.); hot9198@yonsei.ac.kr (Y.C.)

**Keywords:** flexible electronics, nano-fabrication, top-down approaches, bottom-up approaches

## Abstract

Recent progress in fabricating flexible electronics has been significantly developed because of the increased interest in flexible electronics, which can be applied to enormous fields, not only conventional in electronic devices, but also in bio/eco-electronic devices. Flexible electronics can be applied to a wide range of fields, such as flexible displays, flexible power storages, flexible solar cells, wearable electronics, and healthcare monitoring devices. Recently, flexible electronics have been attached to the skin and have even been implanted into the human body for monitoring biosignals and for treatment purposes. To improve the electrical and mechanical properties of flexible electronics, nanoscale fabrications using novel nanomaterials are required. Advancements in nanoscale fabrication methods allow the construction of active materials that can be combined with ultrathin soft substrates to form flexible electronics with high performances and reliability. In this review, a wide range of flexible electronic applications via nanoscale fabrication methods, classified as either top-down or bottom-up approaches, including conventional photolithography, soft lithography, nanoimprint lithography, growth, assembly, and chemical vapor deposition (CVD), are introduced, with specific fabrication processes and results. Here, our aim is to introduce recent progress on the various fabrication methods for flexible electronics, based on novel nanomaterials, using application examples of fundamental device components for electronics and applications in healthcare systems.

## 1. Introduction

Currently, standard electronic devices require multi-functional platforms in a limited substrate area. To keep in pace with the demands of the device users, nanofabrication processing for flexible electronics [1,2,3,4,5] has been deeply studied in a variety of fields, such as multifunctional energy storage devices [6,7,8], wearable devices for human healthcare [9,10,11,12], and displays [13,14,15], with great practicality and reliability.

More specifically, the approaches to device fabrication with mechanical flexibility can be divided into two dominant processes, namely, top-down [16,17,18,19] and bottom-up [20,21,22] approaches. The main differences between the top-down and the bottom-up approaches are the mechanisms and methods for the initial state, such as from the bulk-structure to the desired flexible device for top-down, or the complete opposite method for bottom-up. Here, we introduce ultrathin nano-material structures and fabrication methods that allow assemblies of heterogeneously integrated functional materials onto soft substrates, with all of the active components maintaining excellent electronic functionality. In addition, we focus on well-designed unconventional biomedical devices using the top-down approach and multifunctional flexible sensors from the bottom-up approach, including a flexible graphene transistor, photonic device, MoSe_2_ transistor, and light emitting diode (LED) for the initial components. These flexible and stretchable electronic systems, with performances that reach or exceed the levels of conventional electronic systems, are classified. This review summarizes some recent progress in the field of micro- and nano-electronics and showcases the practical applications of the novel electronic devices.

## 2. Novel Devices Designed by Top-Down Nanofabrication

### 2.1. Introduction to the Top-Down Approach

The top-down approach is a process where the structure is removed from a larger substance. In particular, in the area of macro-electronics, the top-down approach is more desirable because various materials can be produced by conventional lithographic processes [23,24] and etching techniques [25,26,27]. The lateral dimensions of this method can range from tens of nanometers to the millimeter scale. Additionally, freestanding single-crystal sheet types can be created from wafers by employing an embedded release layer so as to yield flexible systems. Thus, nanostructures are synthesized by etching the layers on a substrate. However, it is impossible to conduct the entire procedure on a flexible substrate, because certain processes, such as the doping process and chemical vapor deposition (CVD), require high temperatures that significantly deform flexible substrates [28]. To resolve this issue, a transfer-printing process and a method of releasing from the rigid substrate are integrated to form flexible electronics [29,30,31,32,33,34,35,36]. Here, various flexible electronic devices that are fabricated using the top-down approach, are introduced.

### 2.2. Transfer-Printed Graphene Lines for Flexible Transistor

**Figure 1:**
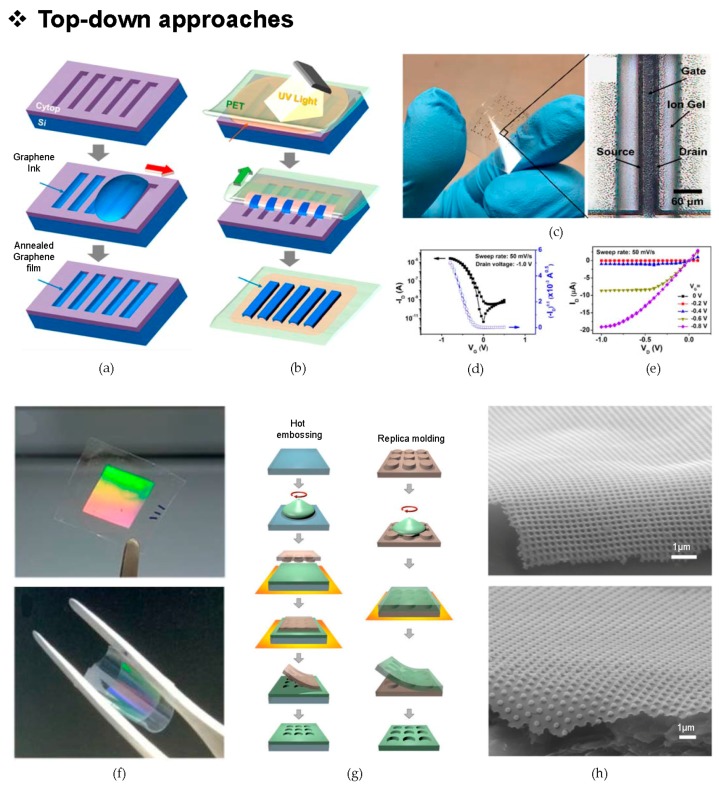
Graphene lines-based flexible transistor/hydroxypropyl cellulose (HPC) photonic thin film with a hexagonal nanopillar structure: (**a**) a schematic of the fabrication of the Cytop/Si mold; (**b**) simple procedures for transferring graphene lines from the Cytop/Si mold to the polyethylene terephthalate (PET) flexible substrate; (**c**) an image of flexible electrolyte-gated transistor (EGT) arrays, the magnified picture shows the composition of each transistor; (**d**,**e**) the transfer and output characteristics of the graphene electrodes, Reproduced with permission from 2017 ACS NANO [37]; (**f**) images of an HPC photonic crystal (top) and its mechanical flexibility with a free-standing property of the design (bottom); (**g**) schematics of two fabrication processes to make HPC photonic films-hot embossing and replica molding methods. The blue-colored slide indicates the glass substrate, the green one for HPC, and the brown one for hard polydimethylsiloxane (h-PDMS); (**h**) special hexagonal nanopillar images obtained by scanning electron microscopy (SEM) in the lateral view. Paper substrates are used to imprint the predesigned nanopattern. Reproduced with permission from 2018 Nature [38].

Recent work demonstrates that graphene-based electrolyte-gated transistors (EGTs) can be introduced as a platform via transfer printing with a silicon stencil, to produce graphene lines [37]. The transfer printing process, which is conducted with a viscous graphene-flake ink, enables transistors to maintain their great electrical performance, while achieving a high printing resolution with flexibility and a scalability at low cost; such results are not easily achieved by the conventional rigid substrate-based fabricating processes. Figure 1a shows the schematics of the detailed fabrication steps, starting with the spin-casting of a Cytop film on the micro-lithographically patterned Si wafer to design a mold. Then, graphene ink is squeezed through the line-holes of the Cytop/Si stencil, forming five groups of graphene lines using the differences in surface energies and wetting properties at the interface of the mold and ink. After the annealing of the graphene film, the graphene lines are ready to be transferred onto a flexible substrate. Figure 1b shows the specific procedures for transfer printing graphene lines onto a polyethylene terephthalate (PET) substrate. A liquid Norland Optical Adhesive (NOA73, Norland Products, Inc., Cranbury, NJ, USA) is coated on the graphene lines, and then the ultraviolet (UV) light is illuminated at the backside of the O_2_-plasma-treated PET substrate. The graphene lines are subsequently peeled-off from the Cytop/Si mold by the UV-cured NOA73 adhesive layer. Figure 1c shows the flexible aerosol-jet printed transistors, which are fabricated on the basis of poly(3-hexylthiophene) (P3HT, Sigma-Aldrich, Saint Louis, MO, USA) as the semiconductor, ion gel for the gate dielectric and graphene lines for the source-drain contact pads. Figure 1d,e present the transfer characteristics and output characteristics of the device, respectively. These two graphs show the operation of the transistor with negligible drain current (I_D_) hysteresis and clear I_D_ saturation. This unconventional fabrication process of graphene transistors provides benefits for circuit design, and by increasing the packing density with transfer printing, assures compatibility for the scalable manufacturing of the flexible devices.

### 2.3. Flexible Photonic Device with Hexagonal Structures

Flexible photonic and plasmonic devices based on eco-friendly hydroxypropyl cellulose (HPC) are introduced in Figure 1f [38]. In general, when the nanoscale celluloses exist in the form of fibers, they may not be seen as white in color, but are seen as possessing a transparent or iridescent property. This optical property can be arbitrarily adjusted by the structure of the particles via either the amorphous state or the single-crystalline state. Using the special chiral behavior [39,40,41,42] that is possessed by an HPC solution, a simple nanostructured design provides an enhanced photoluminescence and the potential of a HPC plasmonic membrane in the role of an optical device. The core fabrication process is focused on exploiting soft lithography [43,44,45,46,47] rather than using conventional photolithography or a growth method. Soft lithography realizes a cost-effective process with large-area patterning, ensuring enhanced reproducibility. Figure 1g shows two specific approaches that are used to fabricate photonic HPC films. The first one is the hot-embossing process. Spin-casted HPC on a rigid substrate is heated, which is followed by a hard polydimethylsiloxane (h-PDMS) compound-based patterning. Then, the HPC photonic film is transferred onto the flexible substrate in order to complete the device fabrication. The second method is to use a replica molding process. On the surface of an h-PDMS mold, the HPC solution is poured and dried by applying heat. Finally, a free-standing, flexible photonic HPC film is obtained by peeling from the PDMS mold. The complete structures that are acquired from both methods are nearly identical, but the hot-embossing process presents a product with a better optical property. After thdepositing and patterning a metal on the HPC photonic film, the fabrication of the HPC film can be completed. Figure 1h shows the scanning electron microscopy (SEM) image of the plasmonic HPC film that has been patterned with arrays of a hexagonal lattice structure. This unique nanostructure upgrades the photoluminescence by maintaining its iridescent property with amplified optical extinction spectra.

### 2.4. Novel Biomedical Electronics—Piezoelectric Probes for Biopsy Diagnosis

Flexible electronics that are fabricated by top-down approaches can be directly applied to medical applications because of the absence of a mechanical mismatch between the flexible electronics and the organs or tissues of the human body. The contents that are to be introduced next are new bio-integrated devices, which can be widely applied in medical treatment/diagnosis. Here, we introduce the devices that can be formed by lifting off a thin layer from a temporary substrate using chemical dissolution [48,49], then, by transfer printing the completed electronic systems from a rigid substrate to a flexible substrate.

**Figure 2:**
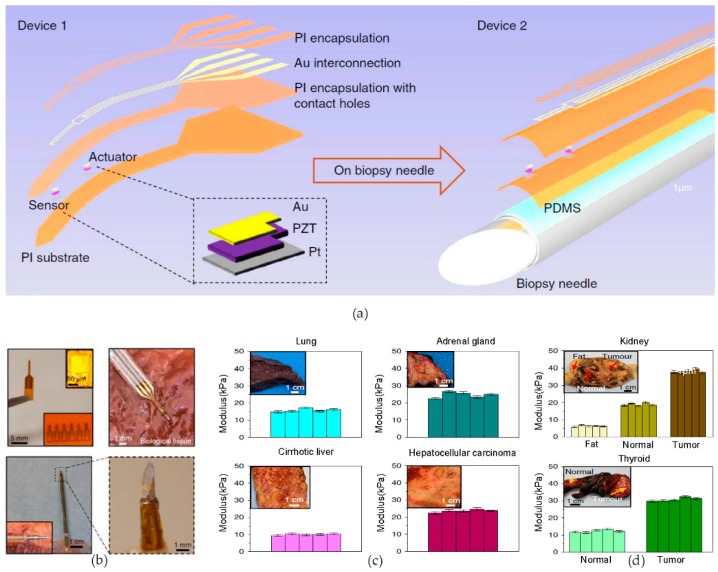
Flexible, piezoelectric tissue modulus recording probes: (**a**) Illustrations of two main piezoelectric material lead zirconate titanate (PZT)-based modulus probes, the free-standing device (left) and the conformally attached device on a biopsy needle (right); (**b**) pictures of self-standing devices (top) and the device on a biopsy needle (bottom), an inset picture (top, upper-right) designates a sensor/actuator pair, and the other inset picture (top, down-right) shows a row of completed designs. The magnified picture (bottom) shows specific actuator/sensor sites; (**c**,**d**) the measured modulus results for human tissues in a variety of organs using the PZT-based probe, the inset pictures of the upper-left side of the respective graphs show the optical images of organs in which modulus sensing is conducted. Reproduced with permission from 2018 Nature [50].

In the first instance, to distinguish the normal tissue from abnormal, a highly flexible microscale piezoelectric device is developed [50]. Exploiting the alteration of the mechanical properties of tissue modulus in accordance with lesion expression [51,52,53,54,55], a novel minimally invasive probe offers quantitative agreement with clinical insights. The magnified schematic views of the specific structures are shown in Figure 2a. The thin, photolithographically patterned and defined triple-layer membrane, which is composed of Au/Cr, the piezoelectric material lead zirconate titanate (PZT), and Ti/Pt, is used as both an actuator and a sensor, simultaneously. Each of the components is transfer-printed onto the polyimide (PI) substrate from the donor wafer, regardless of the structure, in the form of either a free-standing device or integrated on a biopsy needle. After encapsulating the triple layer with PI, another layer of PI is used for encapsulation, after the deposition of the interconnecting Au lines. Figure 2b shows the images of the freestanding probe that has been placed into a biological tissue environment and the device has been wrapped around the surface of a conventional biopsy needle. The bottom right magnified image in Figure 2b designates the point of the actuator/sensor region on the needle. The fundamental device operation is based on the piezoelectric effect [56,57,58,59,60]. When the device achieves conformal contact with the tissue interface, the induced potential results in the piezoelectric strain at the actuator. The tissue that exhibits the deformation transfers this force to the sensor, and then, a portion of the strain force is changed into a certain value of voltage, thereby realizing the measurement of the tissue modulus. Figure 2c shows the results of the tissue modulus determination that has been conducted in human organs, such as fresh lung, adrenal gland, and fresh cirrhotic liver, as well as in a hepatocellular carcinoma. As estimated, Figure 2c provides the information that the cirrhotic liver maintains an average modulus of approximately 10 kPa, while the cancerous hepatocellular tissue reaches a peak modulus of approximately 23 kPa. The modulus graph in Figure 2d also shows the different modulus values that have been acquired between the fresh tissue and the abnormal tissue regions in the human thyroid and a formalin-treated kidney. In this example, the devices offer an underwork for the modulus-measuring platforms for biopsy guidance, based on magnetic resonance elastography [61,62,63,64,65].

### 2.5. Novel Biomedical Electronics—Implantable, Soft Electronic Systems for Optical Stimulation

**Figure 3:**
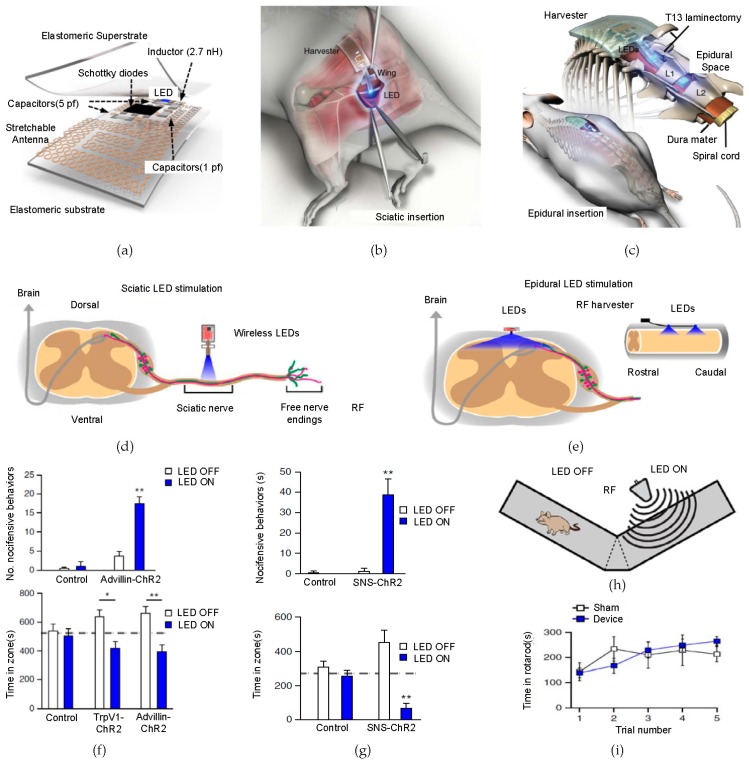
Fully implantable, flexible system for a wireless optogenetic stimulator with related experiments. (**a**) Schematic of a fully implantable, flexible electronic system for a wireless optogenetic stimulator; (**b**) images of the sciatic insertion surgery to implant the completed device into the spinal cord. The wing-shaped light emitting diode (LED) extension site passes from the gluteus maximus to the sciatic nerves; (**c**) a similar implantation is conducted at the epidural space in the spinal cord; (**d**,**e**) an illustration of the nociception path with an LED stimulator in a sciatic nerve and epidural space; (**f**,**g**) graphs of number of adverse behaviors/time in y-maze observed by the Advillin-ChR2-treated rodents in accordance with the LED stimulation in the sciatic nerve and epidural space; (**h**) an illustration of the experimental y-maze. Using radio-frequency (RF) signal, pathways are separated by LED on/off environments; (**i**) the graph of a rotarod performance result. This graph shows that motor activity does not change the entire performance of the device. Reproduced with permission from 2015 Nature [77].

The top-down approach for nanopatterning can also be extensively applied to electrophysiology mapping [66,67,68,69] and electrical/optical stimulating systems [70,71,72,73,74,75,76]. Recently, opto-genetics have been extensively studied because of its delicate controllability of neural activity by selectively modifying channel-rhodopsin-treated genes with light. An entirely seamless implantable optoelectronic device, which lets axons react directly in accordance with light stimulation, is introduced in Figure 3a [77]. The radio-frequency (RF) harvesting unit rectifies the signals that have been received from the transmitter and routes the output current for the optical energy (LED). Then, an interconnected serpentine-structured Ti/Au antenna layer diminishes the resonant frequency while maintaining a wide-bandwidth requisite for efficient energy harvest at certain frequencies. The fabrication process for the device is initiated from the spin-casting of polyimide (PI) onto polymethyl methacrylate (PMMA) that has been coated on a glass surface. Then, a bilayer of Ti/Au is deposited by an e-beam evaporator and it is photolithographically patterned into the serpentine structure to form an antenna. After encapsulating the completed device with polydimethylsiloxane (PDMS), this component is immersed in an acetone solution so as to dissolve the PMMA layer. Finally, the thin free-standing composite optogenetic device is released from the glass, which results in a soft, flexible property. This flexible, stretchable state optimizes the conformal contact between the surface of the tissue and the device. An optogenetic control experiment is conducted, mainly in two parts, namely, underneath the gluteus maximus muscle and in the epidural space in the lumbar spinal cord of mice, as shown in Figure 3b,c. In order to determine the device functionality as an optogenetic platform, an experiment of Ch R2 activation for the nociceptive reaction of mice with spontaneous pain expression, following space abhorrence, is conducted. Figure 3d,e demonstrates the nociceptive pathways via the LED stimulation of the device that is fully implanted at the sciatic nerve and in the epidural space in the Ch R2 expressive mice, respectively. A rotarod test is conducted so as to identify the motor activity of the experimental rodent that has been affected by the performance of the LED stimulator (Figure 3i). The result proves that there is no change in the balance of the device. For specific experimental data, both mice expressing the Ch R2 and the control units are introduced to a y-shaped maze, where the mice can arbitrarily move to either side of the maze. In particular, at the left side of the passage in the y-shaped maze, there is an RF antenna that turns on the implanted LED, as illustrated in Figure 3h. Figure 3f,g show the number of nociceptive expressions and the trend of space abhorrence checking times of staying by the Advillin-Ch R2 for sciatic nerve-implanted mice, SNS-Ch R2 in the epidural space of spinal cord of mice, and the control units, according to the LED illumination. This result shows the expected correlation for stimulation, which presents the potential for the clinical device as a novel optogenetic therapy system [78,79] with great advantages, such as physical tether-free and external-feature-free characteristics.

### 2.6. Novel Biomedical Electronics—Cardiac Patches for Electrical Sensing, Stimulation, and Drug Delivery

**Figure 4:**
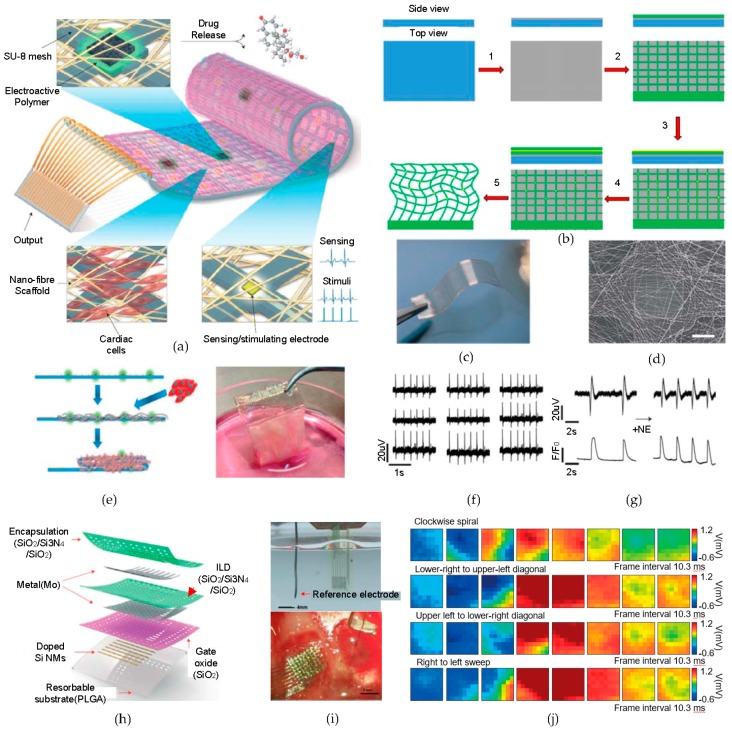
Multifunctional electronic system with biomaterial-based three-dimensional (3D) scaffold cardiac patches/multiplexed bio-resorbable silicon-based design for brain mapping. (**a**) Designed cardiac patches for recording electrical tissue activity/providing electrical stimulation/spatial releasing of biochemical factors; (**b**) the detailed fabrication process of the cardiac patches, the upper picture is the side view and the bottom picture is the top view of the device. The components include the temporary silicon wafer (blue), nickel layer (gray), SU-8 resist (green), and Au (yellow). (1) Deposition of a 20 nm nickel relief layer; (2) photolithography of SU-8 for the mesh structure; (3) patterning and Cr/Au deposition followed by the metal lift-off process and titanium nitride (TiN) layer deposition; (4) coating the substrate with a uniform layer of SU-8, followed by photolithography, and curing and releasing the device from the substrate by etching the nickel layer with nitric acid; (**c**) a picture of a flexible device comprising 32 gold electrodes with a mesh of SU-8; (**d**) an SEM image of larger-sized electrodes in the cardiac patches with nanofiber-based 3D scaffolds; (**e**) a schematic of foldable patches with a conformal cell culture (left). The brown dots are electrodes that are used to release chemical factors, the gray serpentine lines are compounds of the 3D biomaterial scaffolds, and the pink lines are seeded cardiac cells. An image of the device in cell cultures (right); (**f**) the results of electrical signals from cardiac tissues recorded by nine gold electrodes; (**g**) images of increased signal frequency after treating with a neurotransmitter(norepinephrine) on the tissue (top) and integrated recordings through calcium imaging (bottom). Reproduced with permission from 2016 Nature [80]; (**h**) an enhanced schematic of the composition of an actively multiplexed brain-mapping sensor based on the highly doped SiNM; (**i**) a bio-resorbable device in phosphate buffer saline (PBS) with a reference electrode (top) and a magnified image of the implanted device at the left side of a rat hemisphere (bottom); (**j**) epilepsy signal-traced brain mapping results acquired by electrode channels from the device with various spikes. Reproduced with permission from 2016 Nature [81].

The method of releasing an active device from a rigid substrate to obtain device mechanical flexibility is efficiently used in multifunctional cardiac patches (Figure 4a) [80]. These novel cardiac patches are composed of three main parts, namely, an electronic system for sensing and stimulating, electroactive polymer-deposited electrode sites for chemical factor diffusion to control cellular function, and three-dimensional (3D) dense nanofiber scaffolds in the cardiac cell environment. A detailed fabrication process is illustrated in Figure 4b. The device fabrication begins with depositing nickel as a relief layer onto a silicon substrate. Then, the spin-casting and curing of SU-8 photoresist on the surface of the Ni layer occurs, which is followed by the Cr/Au metal lift-off process. Subsequently, a titanium nitride (TiN) layer is deposited on the gold pads by sputtering, so as to increase their surface area. The passivated components of the design are released from the previous silicon substrate, and the remaining free-standing, flexible structure is obtained by etching the Ni relief layer using nitric acid. Figure 4c is an image of the flexible device, which consists of 32 electrodes with an SU-8 mesh structure.

Fascinating cardiac cell cultures are optimized by the stitched-structure-like nanofiber-based biomaterial scaffolds. As shown in Figure 4d, the integration of the biomaterials gives the potential for on-demand drug release to control tissue functions without interfering with the seeding of cardiac cells. The schematic representation and picture of a device that has been cultivated in the cardiac site in Figure 4e indicates the process for the cell culture and the foldable scaffold structure with reliable cell viability. Electrical signal recordings that have been acquired by cardiomyocytes are done from nine gold electrodes in the device, shown in Figure 4f. As a result, all nine of the electrodes show similar results to the signals that have been generated by the cardiomyocytes. Figure 4g presents the results of the cardiac patch sensing for a signal frequency twice as high as that of administering norepinephrine and the integrated quantification recording, through calcium imaging.

### 2.7. Novel Biomedical Electronics—Bio-Resorbable, Ultraflexible Electronic Device for Transient Brain Mapping

In addition to the ability to sense temporary electrophysiological signals from the brain, a multiplexed bio-resorbable array of silicon transistor is introduced in Figure 4h [81]. This particular device is implanted over the cortical surface of a rat brain and functions for a programmed time period, resorbing in the body of the rat. Since the device only consists of biocompatible and biodegradable materials, a secondary surgery to extract the device is not required. After recording the brain activities for a predefined period of time, the device gradually dissolves in the body. The device is based on an actively multiplexed array with Si nano-membrane (SiNM) and N-metal-oxide-semiconductor (NMOS) transistors, which serve as the sites of the electrodes. The SiO_2_ layer serves as the gate insulator, and the Mo serves as contacts for the source, drain, and gate. Two sets of triple layers of SiO_2_/Si_3_N_4_/SiO_2_ provide interlayer dielectric and encapsulation barriers. The fabrication begins with a silicon on insulator (SOI) wafer, which is doped with phosphorous to form the source and drain for the NMOS transistors. Then, the Si-NM layer is separated from the bulk Si wafer by dissolving a box SiO_2_ layer, using hydrofluoric acid (HF). The Si-NM is then transfer-printed onto a thin PI/PMMA/Si substrate using a PDMS stamp, followed by the isolation of the SiNM, depositions of SiO_2_ as the gate dielectric, and Mo for the source, drain, and gate contacts, to form the active matrix. The device is encapsulated and isolated with trilayer of SiO_2_/Si_3_N_4_/SiO_2_. Additionally, another layer of Mo is deposited via holes that are separating the columns and rows to form interconnects. Finally, the encapsulation of the device with another layer of a dilute PI and the formation of mesh structures by photolithography leads to transferring the entire active layer to a bio-resorbable substrate. After dissolving the PMMA layer using acetone to release the entire device, the mesh is consequently transferred onto the surface of the bio-resorbable poly-(lactic-co-glycolic acid) (PLGA) substrate to finalize the device fabrication. Soak testing is conducted in a phosphate buffer saline (PBS, pH 7.4) solution, as shown in Figure 4i (top). Figure 4i (bottom) shows an image of a conformally implanted bio-resorbable array in the left hemisphere and the control electrode in the right hemisphere of an anaesthetized rat brain, for in vivo recording. A series of eight movie frames from each epileptic spike activity, which are induced by applying picrotoxin, are shown in Figure 4j. The electrical mapping results of different forms of spikes (e.g., clockwise spiral, lower right to upper-left diagonal, upper left to lower-right diagonal, and right to left sweep) that have been recorded from the electrodes clearly demonstrate the spatio-temporally resolved neural propagation, thereby ensuring the potential for device use in the clinical arena or health-care systems.

## 3. Novel Devices Designed by Bottom-Up Nanofabrication

### 3.1. Introduction to Bottom-Up Approach

The components of flexible electronic devices at the nanoscale can be fabricated using the stacking of atoms or chemicals from the bottom to the top, referred to as bottom-up approaches. Such methods have distinct advantages in certain aspects compared to top-down approaches. The device fabrication process can be controlled precisely using bottom-up approaches and can be achieved at a relatively lower temperature, so that there is no risk of substrate deformation as a result of the applied heat [82]. There are various approaches to fabricate device components with novel materials using bottom-up approaches, such as growth methods [83,84,85,86,87], assembly methods [88,89,90], and chemical vapor deposition (CVD) [91,92,93,94,95,96]. Here, we introduce various applications for flexible electronic devices that have been fabricated by bottom-up approaches, such as a flexible transistor based on a MoSe_2_ film, flexible light emitting diodes (LEDs) [93], a flexible touch screen [90], flexible strain sensors [83,88,89,97], and flexible temperature sensors [91].

### 3.2. Flexible Transistors Fabricated by Modified Chemical Vapor Deposition (mCVD)

**Figure 5:**
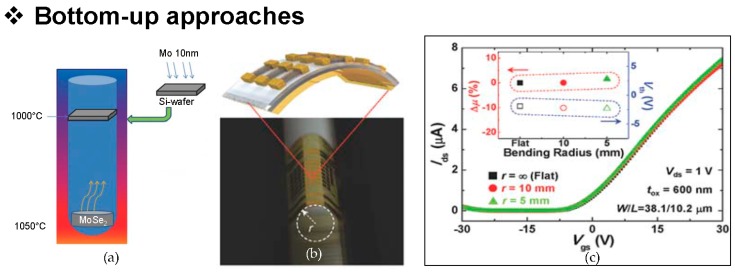
High-mobility transistors based on chemical vapor deposition (CVD)-grown MoSe_2_; (**a**) schematic of synthesis of MoSe_2_ film with modified CVD method; (**b**) optical image of bended MoSe_2_ transistor; and (**c**) I–V characteristic of unbent and bent (up to 5 mm of radius) MoSe_2_ transistor. Reproduced with permission from 2016 Advanced Materials [92].

As the most basic and necessary active device components in modern electronics, transistors have been developed with efforts focusing on reducing their size and power consumption. Moreover, researchers seek to find alternative materials for Si, which is mainly used as the material for conventional electronic devices, because the limitations in fabrication to achieve smaller physical dimensions give rise to low device packing densities. As a result of the mechanical rigidity of conventional transistors, there are limitations in applying them to flexible electronic devices. Therefore, flexible transistors have been widely studied. Here, transistors that are based on MoSe_2_ films have been developed, which demonstrate high mobility and almost no difference in electrical performance, according to the I–V curves that have been obtained at different bending radii [92]. With the conventional chemical vapor deposition (CVD) process, large-scale MoS_2_ films have poor electrical properties, such as field-effect mobilities lower than 15 cm^2^·V^−1^·s^−1^ [98,99,100,101]. To form a MoSe_2_ device layer with a high field-effect mobility, a modified CVD (mCVD) process that uses the polycrystalline compounds of MoSe_2_ as the precursor to directly synthesize the product on SiO_2_ is used. (Figure 5a) On the polyethylene terephthalate (PET) substrate, Ti/Al gate electrodes were deposited by electron beam evaporation and the dielectric material (SU-8 2000.5, MicroChem, Newton, MA, USA) was spin-coated. Then, a single crystalline multilayer of MoSe_2_ flakes were transferred and the source-drain electrodes were defined to form flexible MoSe_2_ transistors. High-mobility transistors based on a crystalline MoSe_2_ film that have been grown on insulating SiO_2_ can be fabricated with a field-effect mobility of 121 cm^2^·V^−1^·s^−1^. Figure 5b shows the fabricated flexible transistors with a bending radius of ‘r’. Figure 5c shows the transfer curves of a MoSe_2_ transistor in different bending regimes. The black, red, and green curves represent the MoSe_2_ thin-film transistor (TFT) without bending and with bending, with radii of 10 mm and 5 mm, respectively. As a result, no significant changes or shifts in the transfer curves are found while changing the bending radius of the device. Therefore, the MoSe_2_ TFTs that are fabricated by the modified CVD process are robust to induce mechanical stress, while maintaining their electrical performance.

### 3.3. Flexible Light Emitting Diodes (LEDs) and Flexible Touch Screen

**Figure 6:**
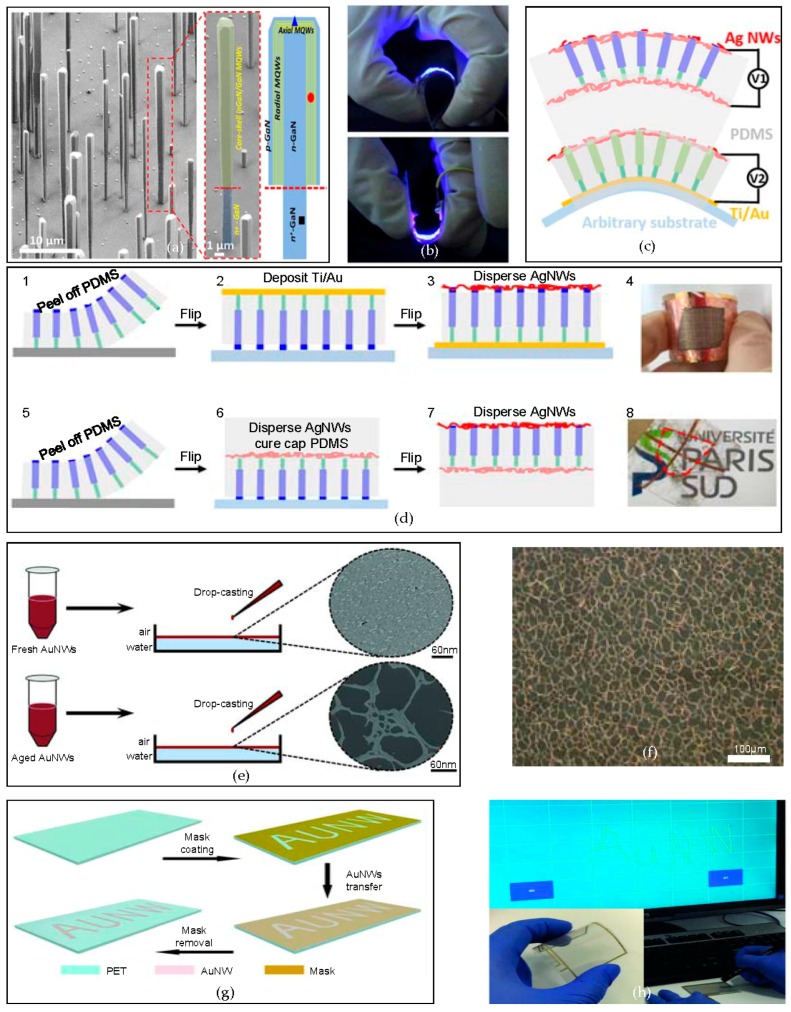
Flexible light emitting diodes based on vertically grown nitride nanowires/transparent and flexible nanomesh via self-assembly of ultrathin gold nanowires to fabricate a flexible touch panel. (**a**) An scanning electron microscope (SEM) image of a grown individual nanowire obtained with a tilt angle of 45°; (**b**) The flexibility of the fabricated nanowire blue LED; (**c**) A schematic of the bi-layer flexible LED with a blue LED for the top layer and a green LED for the bottom layer; (**d**) A schematic of the fabrication process for the semitransparent LED (1–4) and fully transparent LED (5–8), Reproduced with permission from 2015 Nano Letters [93]; (**e**) A transmission electron microscopy (TEM) image of a gold nanowires (AuNW) nanomembrane fabricated by a fresh AuNW solution and an SEM image of a AuNW mesh film fabricated from an aged AuNW solution; (**f**) An optical image of the AuNW mesh film with pores fabricated by the self-assembly method from an aged AuNW solution; (**g**) A schematic of the patterning of the AuNW mesh film on PET; and (**h**) A flexible touch panel fabricated using the AuNW mesh film. Reproduced with permission from 2016 Advanced Electronic Materials [90].

Flexible light-emitting diodes (LEDs) open the path to conformal displays on complex curvilinear objects [102,103,104], healthcare devices [105,106], optoelectronic systems [107,108,109], instrumented surgical gloves [110], etc. Nanowires is a well-known material that is used to fabricate flexible LEDs. Among the various types of nanowires, nitride nanowires have remarkably good optoelectronics properties with excellent resistance to mechanical deformation [111]. Here, flexible LEDs based on nitride nanowires are introduced [93]. In particular, fully flexible blue LEDs using nanowires with a core/shell of InGaN/GaN that are grown via metalorganic chemical vapor deposition (MOCVD) have been demonstrated, as shown in Figure 6a. The fabricated blue LEDs (Figure 6b) show no degradation in light brightness, down to a bending radius of 3 mm and without encapsulation, while the conventional flexible LEDs require encapsulation barriers to protect the LEDs. In addition, two-layer bicolor flexible LEDs based on nanowires emitting blue and green light at the same time, have been demonstrated (Figure 6c). To fabricate the semitransparent green LED layer, which is the top layer of the two-layer bicolor flexible LED, on a thin metal shell of Ni/Au, GaN nanowire arrays are embedded in PDMS and are peeled off. Then, the whole layer is flipped to deposit Ti/Au, which serves as an arbitrary substrate. Since the AgNWs provide a high electrical conductivity, silver nanowires (AgNWs) are dispersed on the side opposite to the deposited Ti/Au (Figure 6d, 1–4). For the fabrication of the fully transparent blue LED, the fabrication process is similar to that of semitransparent LED. However, instead of the Ti/Au deposition, optically transparent AgNWs are dispersed on both sides of the PDMS-encapsulated layer (Figure 6d, 5–8). The fabricated two-layer flexible LED shows no significant performance degradation in I–V and electroluminescence (EL) characteristics at 3.5 mm and 2.5 mm bending radii, with repeated bending cycles.

The touch screen is another application of flexible electronic devices that have been fabricated via bottom-up approaches. Here, a solution-based self-assembled nanomesh, using aged gold nanowires (AuNWs), is fabricated [90]. As Figure 6e shows, keeping fresh AuNWs for 12 h before drop-casting forms aged AuNWs. The aged AuNWs self-assemble into bundles creating a continuous nanomesh structure with a pore size of 8–52 μm. While the steric hindrance of the fresh AuNWs is stronger than the wire-to-wire van der Waals force that maintains the ordered structure of nanomembrane, the steric hindrance and the van der Waals force balance is destroyed for the aged AuNWs, which creates bundles of AuNWs. As a result of the pores in the nanomesh from the aged AuNWs (Figure 6f), the nanomesh film is transparent, while the nanomembrane that is formed by fresh AuNWs is not. In addition, the nanomesh that is fabricated by the aged AuNWs is electrically conductive with a sheet resistance of 130.1 Ω^−1^. The AuNW nanomesh can be transferred onto the polyethylene terephthalate (PET) with a sacrificial layer of a mask sheet. With the removal of the sacrificial mask by peeling, using tape or washing with ethanol, the patterned AuNW nanomesh is preserved on the PET (Figure 6g). The AuNW nanomesh can be utilized as an array of the pressure sensors for the flexible touch screen, as shown in Figure 6h. The nanomesh film shows a similar level of pressure sensitivity compared to that of commercial products [112].

### 3.4. Novel Flexible Sensors—Strain Sensors

**Figure 7:**
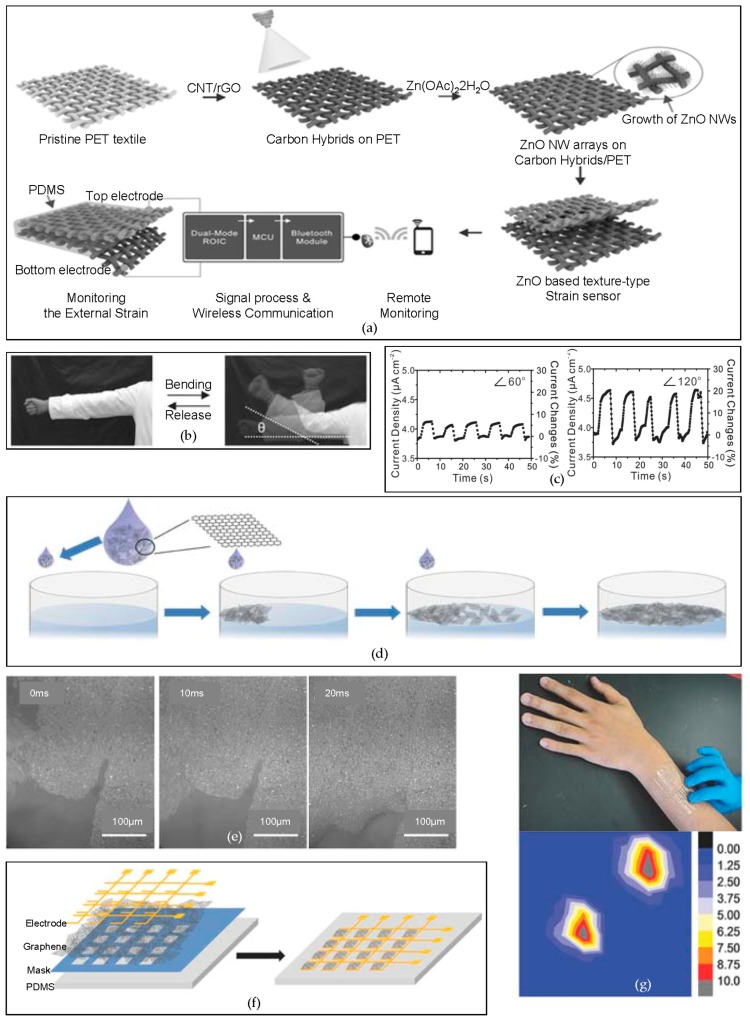
Flexible textile sensor based on a hybrid of carbon materials as the substrate/Strain sensors based on ultrathin graphene films. (**a**) A schematic of the fabrication of a flexible textile strain sensor generated via the growth of ZnO nanowires; (**b**,**c**) The bending and releasing of the cloth made by a flexible textile strain sensor and the corresponding results according to the different bending angles of the arm; Reproduced with permission from 2016 Advanced Functional Materials [83]; (**d**) Graphene flakes forming the ultrathin graphene film (UGF) via the π-π interactions among each other; (**e**) Optical images of the formation of UGF taken in 20 ms by a high-speed charge-coupled device (CCD) camera; (**f**) A schematic of a strain sensor fabricated from the self-assembled UGF; (**g**) A strain sensor applied on the forearm and the real-time response map of pressure applied on the strain sensor. Reproduced with permission from 2016 Advanced Materials [89].

One of the well-known applications of the flexible electronic devices are the flexible sensors. Various types of substrate can be used for flexible strain sensors, from textiles to carbon-based materials. In order to enhance the electrical characteristics of a fabric-based flexible strain sensor, nanowires that are grown on the textile can be used [83]. Herein, a wireless flexible strain sensor is applied on a commercial textile via the assembly of hybrid carbon materials at the nanoscale and piezo-resistive ZnO nanowires (NWs) that have been grown on the commercial textile. The assembled hybrid carbon nanomaterials provide excellent properties in terms of the robust electrical performances against mechanical deformation from bending. On the pristine PET textile, carbon nanotubes (CNTs) and reduced graphene oxide (rGO) are coated so as to provide an excellent growth condition for the ZnO nanowires [113,114]. The coated hybrid carbon nanomaterials provide the continuous conductivity and durability against the externally applied mechanical strain. Then, the ZnO-based texture-type strain sensor is encapsulated with a thin PDMS layer (Figure 7a). Figure 7b,c show the current density change with the bending and the release of the fabricated flexible textile strain sensor. The plots in Figure 7c shows the flexible textile sensor monitoring the repeated bending and release of an arm up to bending angles of 60° and 120°, respectively. The assembled ZnO-3 on the PET substrate textile, which is hybridized with carbon nanomaterials, has a twice-higher gauge factor (GF) of 7.64, than that of ZnO-3 that has been assembled on a plain conventional PET film. This result implies that using the fabric-like structure as the substrate for strain sensors culminates in high-pressure sensitivity and stability at the same time. 

Skin-like electronics, known as e-skins, have been widely studied because of their wide applications, such as health monitoring, medical implantation, and integration of sensors. A wide range of novel materials can be used to fabricate e-skins. Among those materials, graphene is currently one of the most widely used materials to fabricate the active devices [115,116,117,118]. Here, a highly sensitive flexible strain sensor for e-skins is developed using an ultrathin sensitive graphene film that is formed through the self-assembly method [89]. The self-assembly method uses the specific affinity between two molecules [119,120,121]. The molecules that are used to form a self-assembled layer have strong interaction forces, which are noncovalent interactions, so that a monolayer can be formed tightly. Van der Waals [122,123], hydrogen bonding [124], and π-π interactions [125,126] are the representative examples of noncovalent interactions that are used in the self-assembly method. The Marangoni effect [127,128,129], which is the phenomenon of transferring a group because of the difference of a gradient between two different fluids’ surface tensions, is used to form an ultrathin graphene film (UGF). Figure 7d shows the schematic of how the ultrathin graphene film (UGF) is formed by the self-assembly method. Graphene flakes, with a thickness of 2.5 nm, are injected on the surface of the deionized (DI) water, and ethanol is spread on the surface of the DI water. As a result of the Marangoni effect, the ethanol with graphene flakes tend to move toward the high surface tension area, where the DI water is richer than ethanol. Then, the transferred graphene flakes collide and compact together via π–π interactions, forming a large area of UGF, as shown in Figure 7e. The whole process of forming a large-area UGF of 150 cm^2^ takes only 5 s. The advantage of using the self-assembly process that is introduced here, is that the self-assembly process can form the uniform UGF, while the other methods, such as drop-casting and spin-coating, cannot. With the UGF that is formed by the self-assembly method, the strain sensor can be fabricated. As shown in Figure 7f, the UGF is transferred onto the mask, which is attached on the PDMS slab. After removing the mask to form 8 × 8 pixels, the electrodes are transferred to create an electronic skin with the tactile sensors. The UGF that is formed by the self-assembly process shows an extremely high gauge factor (GF) of 1037 at a low strain (2%). The resistance of the individual graphene flakes hardly changes because of the stable crystal structure. However, using the tunneling effect [130] of the overlapped intersection area of the self-assembled graphene flakes, the exceptionally high GF that has been shown in the result, can be achieved. Figure 7g (top) shows the performance of the pressure sensor array, wrapped around the arm. As the strain is applied from the fingers, the spatially resolved pressure sensing can be monitored, as shown in Figure 7g (bottom).

**Figure 8:**
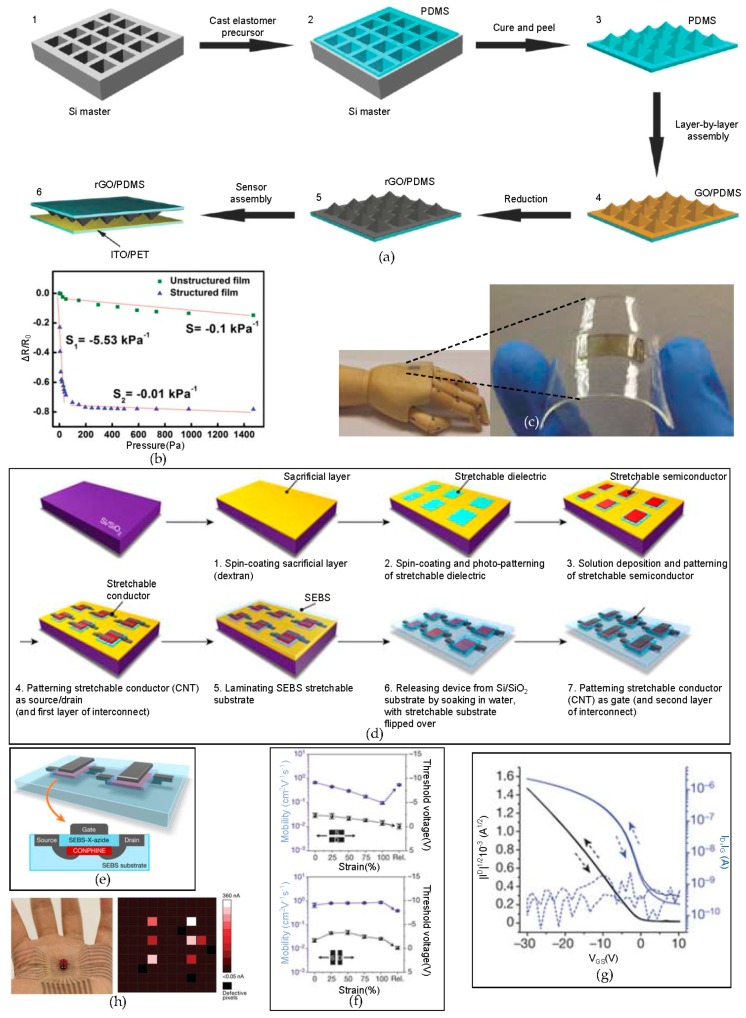
Microstructured graphene arrays for sensitive flexible tactile sensors/electronic skin based on a stretchable transistor array. (**a**) A schematic of the fabrication of a flexible tactile sensor device via the layer-by-layer (LBL) assembly method. An ultrathin graphene film that detects applied pressure is deposited on the PDMS film; (**b**) the pressure response curves of unstructured and structured rGO/PDMS films. The slope of the relative resistance change is −5.53 kPa^−1^ at 100 Pa, which shows very sensitive changes at lower pressures; (**c**) an image of the tactile senor applied on the flexible substrate. Reproduced with permission from 2014 Small [88]. (**d**) a schematic of the fabrication process for arrays of flexible transistors; (**e**) a magnified view of a flexible transistor exhibiting high performance; (**f**) the mobility and threshold voltage changes when applying strain from 0% to 100%; (**g**) the I–V characteristics of fabricated transistors, which have almost no hysteresis; (**h**) stretchable arrays of transistors attached to the human palm with a small artificial lady bug with six legs and the corresponding result from the stretchable sensor. Reproduced with permission from 2018 Nature [97].

Graphene shows a remarkable electrical conductivity, mechanical strength, flexibility, and optical transparency. With these properties, using the graphene and microstructured graphene that has been obtained by the layer via a layer-by-layer (LBL) assembly method [131,132,133,134], an ultrasensitive pressure sensor can be developed. The advantage of the LBL assembly method is the thickness of the LBL assembled layer can be easily controlled through the adjustment of the number of layers and the shape of the LBL assembled layer via uniformly distributed molecules on each layer. As a result ofthe geometrical and self-limiting properties, the surface structure of the ultrathin film that has been formed by the LBL assembly method can be designed in a variety of shapes, while this is not possible with other methods, such as spin-coating or drop-casting [88]. Figure 8a shows the fabrication process of the ultrasensitive flexible tactile sensor. With a KOH-etched Si master mold, pyramid shapes of PDMS can be fabricated. Graphene oxide (GO) from graphite oxide is well suited for the LBL assembly because the GO sheet has abundant negative charges with the hydroxy and carboxyl groups. After depositing the GO sheets on the microstructured PDMS, which improves the sensitivity via structure, the GO layers are treated with hydrazine vapor to form reduced graphene oxide (rGO), which consists of electrically conductive graphene sheets [135,136]. The rGO sheets are sandwiched between the PDMS and an indium tin oxide (ITO)-coated PET film to construct the pressure sensor unit. The tips of the pyramid shapes of the rGO contact the ITO-coated PET film, which increase the sensitivity of the device. Figure 8b shows the relative resistance changes (ΔR/R0) of the pressure changes. The microstructured film is highly sensitive over the unstructured film. The pressure-response curves of the microstructured film can be subdivided into two parts, namely, highly sensitive at lower pressures with a range of 0–100 kPa, and a saturated sensitivity segment with a range above 100 kPa. At the lower pressure range (0–100 kPa), the pressure sensitivity slope is −5.53 kPa^−1^, and at the saturation in the sensitivity range (>100 kPa), the pressure sensitivity slope is −0.01 kPa^−1^. This means the highly sensitive flexible tactile sensor is ultrasensitive at the lower pressure range of 0–100 kPa, with an ultrafast response time of 0.2 ms. As Figure 8c shows, the ultrasensitive at a lower pressure range (0–100 kPa) pressure sensor for e-skin has been developed.

In another example of a strain sensor for e-skin, the arrays of the stretchable transistors are fabricated with components of polystyrene-block-poly(ethylene-ran-butylene)-block-polystyrene (SEBS) as a flexible dielectric, a conjugated polymer/elastomer phase separation induced elasticity (CONPHINE) film as the semiconductor, and spray-coated carbon nanotubes (CNTs) for electrodes [97]. Figure 8d shows the fabrication processes of the arrays of the stretchable transistors. Rigid Si/SiO_2_ is used as the bottom layer to fabricate the arrays, and on the top, water soluble dextran, which is used as a sacrificial layer to separate the active layer from the rigid Si/SiO_2_ layer, is deposited via spin-coating. Then, a stretchable dielectric, stretchable semiconductor, and stretchable conductor (CNTs) are patterned. The SEBS is laminated on to function as the flexible substrate, and the CNTs are patterned to form the gate electrodes. Figure 8e shows the schematic of the formed stretchable transistor. The completed arrays of the stretchable transistors show a slight difference in the carrier mobility when applying external stresses vertically and horizontally (Figure 8f). The average charge-carrier mobility from the array of transistors that were recorded is 0.821 ± 0.105 cm^2^·V^−1^·s^−1^, with the highest value of 1.11 cm^2^·V^−1^·s^−1^, an on/off current ratio of 10^4^, and a low operation voltage of 10 V. In addition, this stretchable transistor array consumes little power, which suggests its potential for use as a self-powered e-skin (Figure 8f,g). Figure 8h shows the stretchable array of the transistors attached onto a palm. The device can detect very small pressure changes with a high spatially resolved pattern and sensitivity, even when an artificial lady bug sits on the device.

### 3.5. Novel Flexible Sensors—Temperature Sensor

**Figure 9:**
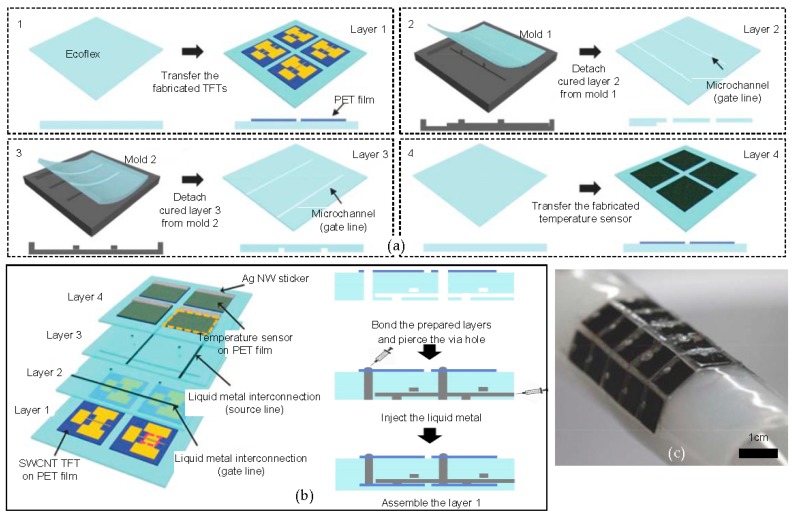
Stretchable temperature sensor array for electronic skin. (**a**) A schematic of the fabrication of the flexible and stretchable active matrix temperature sensor array; (**b**) the vertical schematic view of the sensor (left) and injected liquid metal for gate/source lines; (**c**) an optical image of the bent sensor with a radius of 14 mm. Reproduced with permission from 2016 Advanced Materials [91].

Monitoring the body temperature is important since numerous diseases can be characterized by abnormal changes of body temperature. Therefore, a precise and continuous temperature monitoring device is required. Recent work presents a stretchable and flexible temperature senor in the form of electronic skin, with a stable temperature sensing performance when an external strain is applied [91]. The device consists of a 5 × 5 single-walled carbon nanotube (SWCNT) TFT layer, two liquid metal interconnection layers, and a layer of a 5 × 5 array of temperature sensors. On layer 1, the array of the SWCNT TFTs are on a PET film, having been fabricated via a CVD process and transferred. Layer 2 and layer 3 are microchannel Ecoflex layers, which are formed on a microstructured mold. Layer 4 is the temperature sensing layer with polyaniline temperature sensors on a PET film. Four different layers are electrically connected via a liquid metal that is a compound form of gallium, indium, and tin (Figure 9a,b). Since the device consists of the thin soft elastomer (Ecoflex) and ultrathin active components, the entire device can be stretched up to a strain of 30%, without device fracturing or performance degradation. The completely fabricated temperature sensor can sense both heating and cooling through the normalized resistance change, with respect to the temperature change. There is no hysteresis found in the normalized resistance change for the heating and cooling. Figure 9c shows that the temperature sensor array can be bent up to a radius of 14 mm, while maintaining its electrical performances.

## 4. Conclusions

This review summarizes the most recent developments in the novel fabrication techniques of flexible and stretchable electronics. Modulus-sensing biopsy probes, implantable optoelectronic systems, multifunctional cardiac patches, and bio-resorbable transient brain-mapping electronic designs are introduced in the contents of the top-down approach. Then, Au nanomembrane by self-assembly method for touch screen, flexible textile strain sensing system, graphene-based tactile sensors, stretchable transistor arrays, and temperature sensor designs are also demonstrated in the bottom-up contents. The graphene transistor, photonic devices, MoSe_2_ transistor, and LEDs are also discussed in the introductory contents. Detailed fabrication methods that have been previously mentioned, such as transfer printing with replica molding, transfer and releasing processes, growth, and self-assembly shows that most of the processes are available commercially at low-cost with high quality. These approaches are important because they strongly suggest solutions for high-temperature processes, which are susceptible for the flexible substrate and high scalability in a short time that conventional fabrication processes cannot achieve. Various techniques that have been introduced in this review show the greatest potentials of flexible electronics used as clinical issues, human healthcare system, and multifunctional sensing devices with optimistic practicality and reliability.
